# Obesity in Hypertensive Patients Is Characterized by a Dawn Phenomenon in Systolic Blood Pressure Values and Variability

**DOI:** 10.3390/jcm13020371

**Published:** 2024-01-10

**Authors:** Dawid Lipski, Dorota Marzyńska, Paulina Sytek, Patrycja Rzesoś, Agnieszka Rabiza, Sebastian Żurek, Artur Radziemski, Łukasz Stryczyński, Andrzej Tykarski, Paweł Uruski

**Affiliations:** 1 Department of Hypertensiology, Angiology and Internal Medicine, Poznan University of Medical Sciences, 61-848 Poznan, Poland; dorota.marzynska@student.ump.edu.pl (D.M.); 81512@student.ump.edu.pl (P.S.); patrycja.rzesos@student.ump.edu.pl (P.R.); agnieszka.rabiza@student.ump.edu.pl (A.R.); aradziemski@ump.edu.pl (A.R.); lstryczynski@ump.edu.pl (Ł.S.); tykarski@ump.edu.pl (A.T.); puruski@ump.edu.pl (P.U.); 2 Institute of Physics, University of Zielona Gora, 65-516 Zielona Gora, Poland; s.zurek@if.uz.zgora.pl

**Keywords:** obesity, body mass index, hypertension, blood pressure variability, ambulatory blood pressure monitoring, ABPM

## Abstract

One of the causes of hypertension is excess weight gain, which can also affect the course of this disease. Both the diagnosis and management of hypertension commonly use ambulatory blood pressure monitoring; the results of which correlate more strongly with cardiovascular diseases and cardiovascular death than office blood pressure monitoring. We evaluated blood pressure values and their variability from hour to hour to see if and when they differed between hypertensive patients with and without obesity. The study included 1345 patients who underwent 24 h ambulatory blood pressure monitoring and then were divided into groups according to body mass index and waist circumference. The obtained data were analyzed according to the subjects’ wake-up time, and short-term blood pressure variability parameters were calculated as the mean of the absolute values of the differences between consecutive measurements. The systolic blood pressure in obese subjects was significantly higher between 1 and 5 h before waking than in normal-weighted individuals. In turn, the variability in systolic and diastolic blood pressure was higher with increasing body mass index. The difference in systolic blood pressure values and blood pressure variability was most prominent in the last 5 h of sleep in obese patients.

## 1. Introduction

According to the American Heart Association, 75% of hypertension cases are directly connected to obesity. The incidence of hypertension increases with the increasing incidence of obesity, and these two diseases coexist frequently. Obesity may induce hypertension through several mechanisms, which confirms these epidemiological observations [1,2]. The risk of hypertension not only depends on excess body weight but also on the obesity phenotype, described by the waist circumference.

Twenty-four-hour ambulatory blood pressure monitoring (ABPM) provides information about the blood pressure profile and its variability, unlike office measurements. ABPM parameters (including daytime and nighttime systolic blood pressure (SBP), diastolic blood pressure (DBP), standard deviation, and dipping status) correlate more strongly with the risk of cardiovascular diseases and cardiovascular death than office blood pressure [3,4,5,6,7,8]. ABPM allows for the evaluation of short-term blood pressure variability. Blood pressure variability independently predicts organ damage, cardiovascular events, and cardiovascular deaths [9,10,11]. Jing et al. revealed that, in older patients, elevated blood pressure variability is associated with heart and kidney injuries, independently from blood pressure levels. Increased blood pressure variability serves as a risk factor for left ventricular hypertrophy and decreased renal function [12]. In their study, Sierra et al. demonstrated that short-term systolic blood pressure variability served as a predictor of 1-year mortality in a group of very elderly individuals admitted to the hospital for chronic disease decompensation. Meanwhile, the mean values of systolic and DBP obtained from 24 h measurements did not exhibit a similar predictive capacity [13]. Barochiner et al. reported congruent findings, wherein the most substantial association with hypertension-mediated organ damage, as assessed by parameters such as estimated glomerular filtration rate (eGFR), left ventricular mass index (LVMI), relative wall thickness, ejection fraction, and carotid-femoral pulse wave velocity, was identified in relation to the coefficient of variation (CoV) for SBP and the difference between the maximum and minimum SBP values [11].

In individuals with excess visceral adipose tissue, there are changes in the levels of hormones, called adipocytokines [14,15]. This dysregulation increases inflammation, insulin resistance, endothelium dysfunction, coagulation, sympathetic nervous system (SNS) activity, and renin–angiotensin–aldosterone system activity. Through these processes, obesity contributes to diseases like hypertension, type 2 diabetes, atherosclerosis, dyslipidemia, cardiovascular disorders, and sleep disorders, increasing cardiovascular risk [16,17].

Through ABPM analysis, previous studies have shown that obese individuals present with all blood pressure values higher than lean individuals [18]. Moczulska et al. conducted a comparative analysis between patients exhibiting a body mass index (BMI) ranging from ≥30 kg/m^2^ and <40 kg/m² and those with a BMI ≥ 40 kg/m^2^. Their findings revealed markedly elevated average 24 h, daytime, and nighttime SBP and DBP levels among individuals grappling with morbid obesity. Additionally, a notably increased prevalence of reverse dippers was observed within the latter group of patients, showing a significant difference in occurrence (5.9% compared to 21.3%) [19]. Furthermore, another study emphasized that obesity plays a crucial role in influencing ABPM values, affecting not only individuals with hypertension but also those with conditions like diabetes. In their study, Mathews et al. revealed that individuals with both obesity and diabetes mellitus exhibited significant circadian variations in blood pressure parameters and an elevated prevalence of non-dipping status [20]. Our study aimed to evaluate the differences in the short-term blood pressure variability profile in both treated and drug-naive individuals with essential hypertension in different categories of obesity. In particular, we aimed to determine the exact time of the day or night when the changes between obese and non-obese individuals were the most prominent.

## 2. Materials and Methods

The study included 1345 hypertensive patients referred to the outpatient clinic of the University Clinical Hospital in Poznan in the years 2014–2020. General characteristics of the studied population, i.e., age, sex, categories of BMI and waist circumference distribution, and mean values of office blood pressure and ambulatory blood pressure monitoring, are shown in Table 1.

Arterial hypertension was diagnosed, according to the guidelines of the European Society of Cardiology [21], as values of office SBP ≥ 140 mmHg and/or DBP ≥ 90 mmHg when untreated or when on anti-hypertensive treatment [21,22].

Exclusion criteria were as follows: signs or diagnosis of secondary hypertension, being underage, pregnancy, and breastfeeding. The approval of the local bioethics committee was obtained. Complete medical interviews and examinations were collected for each patient, with an emphasis on the possible causes of secondary hypertension and medications taken. Office blood pressure values were obtained using an Omron 705 IT device by measuring the average value of three consecutive measurements. These measurements were always taken in the sitting position, in a quiet room, after 5 min of rest. An ABPM was performed once using an AND TM-2430 device (A&D, Tokyo, Japan) with the appropriate cuff for each patient. The device recorded the blood pressure every 30 min. Every patient was asked to write down the time he or she went to bed and the time he or she awoke. These real-life times were used in the statistical analysis.

Short-term blood pressure variability parameters for these periods were calculated as the mean of the absolute values of differences between consecutive measurements. We consider this parameter to be a better measure of short-term variability than standard deviation, because standard deviation is not sensitive in situations when there is a high variability from measurement to measurement.

Anthropometric measures (height, weight, and waist circumference) were determined for each patient included in the study. Waist circumference was measured by means of a tape measure with a person standing at the midpoint between the last rib and iliac crest. The protocol was used consistently in every subject. Patients were assigned to two groups: a normal value of waist circumference and an abnormal value of waist circumference. The cutoff points were ≥88 cm for women and ≥102 cm for men according to the National Cholesterol Education Program Adult Treatment Panel III [23].

The BMI was derived using Quetelet’s equation (body mass (kg)/[height (m)]^2^). Patients were divided into 4 groups according to the WHO (World Health Organization)’s BMI criteria [24]:

Group 1: normal patients with BMI < 25 kg/m^2^;
Group 2: overweight patients with 25 ≤ BMI < 30 kg/m^2^;
Group 3: patients with first stage obesity with 30 ≤ BMI < 35 kg/m^2^;
Group 4: patients with second and third stage obesity with BMI ≥ 35 kg/m^2^.

In Table 2, concomitant diseases present in different BMI groups are presented. In Table 3 the medications used in treatment of studied individuals are presented.

We conducted an analysis of the values and variability, hour by hour, for both SBP and DBP in relation to the participants’ declared time of waking up. We have calculated the mean values of pressure and mean values of variability for every 30 min period in different BMI and waist circumference groups. We determined the periods in which the differences between the groups, divided according to BMI and waist circumference, were the highest. The differences between the groups were tested with the Mann–Whitney U test. A *p* value lower than 0.05 was recognized as statistically significant.

## 3. Results

### 3.1. Office Blood Pressure

The mean office SBP in the group of patients with a BMI < 25 kg/m^2^ was significantly lower (*p* < 0.001) compared to each of the other groups. The median office SBP in the individuals with a BMI < 25 kg/m^2^ was 132.5 mmHg, while in the groups with a BMI ≥ 25 and <30 kg/m^2^, BMI ≥ 30 and <35 kg/m^2^, and BMI ≥ 35 kg/m^2^, it was 137.5 mmHg, 141.5 mmHg, and 142 mmHg, respectively. We also found a similar relationship when comparing groups with different waist circumferences; the median office SBP was significantly lower in the group of patients with normal waist circumference (135.0 mmHg vs. 140.0 mmHg, *p* < 0.001). The same relationship was shown for office DBP when comparing participants divided into groups by BMI and waist circumference; in the group with the lowest BMI or normal waist circumference, the office DBP values were significantly lower than those in the comparison group.

### 3.2. ABPM

**Figure 1:**
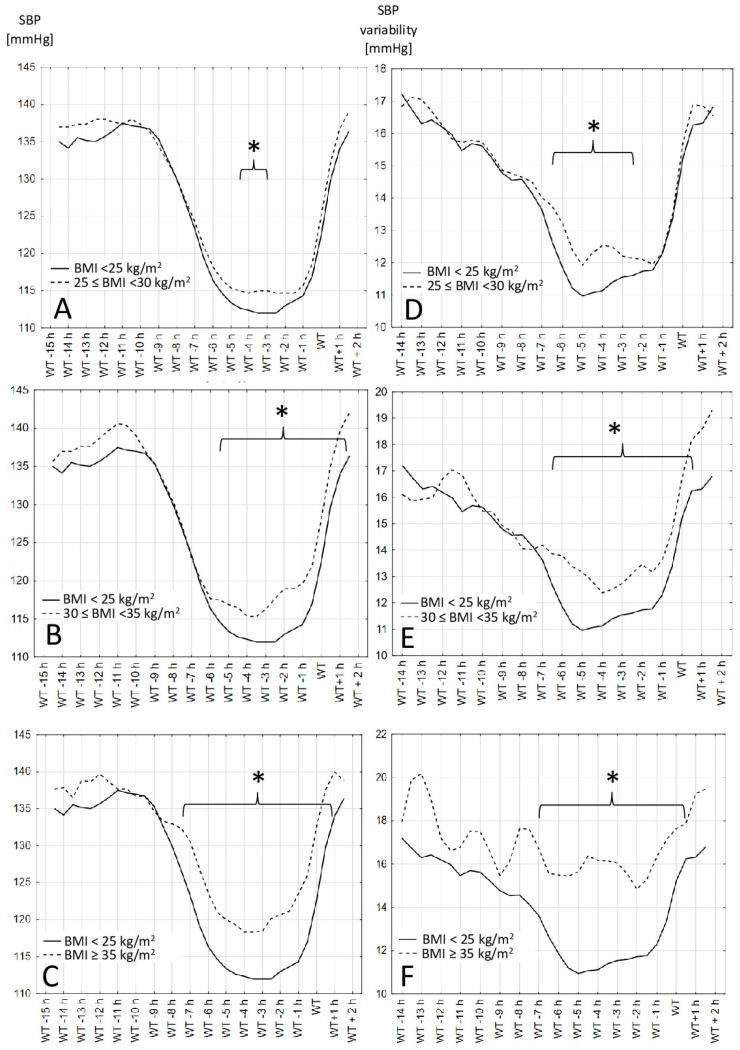
Systolic blood pressure values and variability in different BMI groups. (**A**–**C**)—Systolic blood pressure values in different hours before and after wake-up time in different BMI groups. (**D**–**F**)—Systolic blood pressure variability calculated as the average absolute value of the difference between the consecutive blood pressure recordings in different hours before and after wake-up time in different BMI groups: body mass index, WT: wake-up time. *—differences are statistically significant when *p* > 0.05.

**Figure 2:**
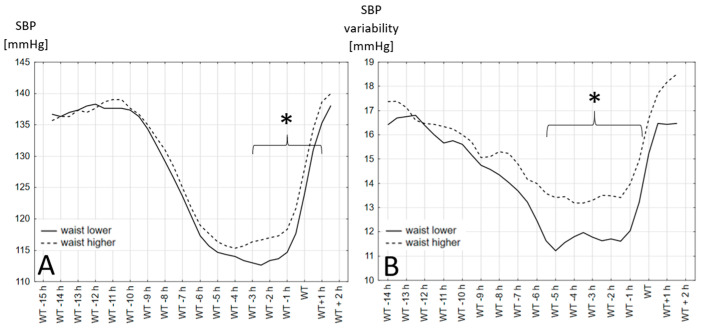
Systolic blood pressure values and variability in two waist circumference groups. (**A**)—Systolic blood pressure values in different hours before and after wake-up time in two waist circumference groups. (**B**)—Systolic blood pressure variability calculated as the average absolute value of the difference between the consecutive blood pressure recordings in different hours before and after wake-up time in two waist circumference groups. *: differences are statistically significant with *p* > 0.05, WT: wake-up time.

The SBP values and variability, measured over 24 h in 30 min periods based on the wake-up time, are shown in Figure 1 (for different BMI groups) and in Figure 2 (for two waist circumference groups). In each figure the mean systolic blood pressure values and variability in each BMI and waist circumference groups are presented. The periods where the difference between presented groups is significantly different are indicated with a bracket and marked with an asterisk.

Both DBP values and variability did not show a significant difference between the BMI and waist circumference groups.

## 4. Discussion

The results show that the time between 1 and 5 h before the declared wake-up time is when the differences between the different BMI and waist circumference groups are the clearest. Obese people have a higher SBP in the period 1 to 5 h before waking up regardless of whether the definition of obesity was based on the BMI or waist circumference. The variability in the blood pressure in the observed groups did not always follow the differences in the blood pressure values.

As we stated in the introduction, our aim was to analyze the blood pressure values and variability hour by hour precisely to find the exact time when they were differentiated between obese and non-obese patients. This is a different aim to other studies. In the articles published prior to ours, the blood pressure values were analyzed for the whole daytime and whole nighttime. The blood pressure variability parameters, e.g., the standard deviation or coefficient of variance, were also calculated for these periods.

Tadic et al., among others, performed ABPM on 127 untreated subjects with hypertension, who they divided into groups according to their BMI values. Their results showed that the nighttime blood pressure (the average of blood pressure measurements from the time when patients went to bed to the time they got out of bed) gradually increased from lean to obese subjects, and the daytime blood pressure (as the average of blood pressure measurements recorded during the rest of the day) was higher in obese subjects than in normal-weight patients. Additionally, the daytime blood pressure variability (measured as standard deviation) was higher in obese (BMI ≥ 30) than in normal-weight subjects, while the nighttime blood pressure variability gradually increased from lean to obese patients [25]. In their study comparing diabetic subjects without hypertension, Mathews et al. found that obese individuals exhibited higher mean nocturnal SBP values than their non-obese counterparts. Conversely, among participants with coexisting hypertension and diabetes, no significant difference was observed in nocturnal SBP values, regardless of obesity status. When categorizing participants based on BMI values (above 25 and below 25), Mathews et al. demonstrated elevated diurnal systolic blood pressure variability (calculated as standard deviation > 10 mm Hg) among obese subjects, with no statistically significant difference noted in diastolic blood pressure variability within these same groups [20]. Palatini et al. also reported a relationship between the BMI of untreated hypertensive participants and the variability in the systolic and DBP during the night [26]. On the other hand, Głuszewska et al. observed a significant reduction in blood pressure variability in hypertensive patients with a BMI ≥ 40 kg/m^2^ after bariatric surgery, which was not found in the normotensive group. Postoperative remission of hypertension was achieved in 41.7% of patients after 6 months [27]. Contrary to the aforementioned studies, Barochiner et al. reported contrasting results. They evaluated home blood pressure monitoring (HBPM) data from treated hypertensive patients, revealing an inverse relationship between BMI and home blood pressure variability. This was calculated either as the coefficient of variation or the difference between the maximum and minimum blood pressure values obtained during HBPM [11]. On the other hand, Chu et al. examined the factors influencing the morning blood pressure surge (MBPS) in untreated hypertensive individuals. They calculated MBPS as the difference between the morning SBP (the average of the four 30 min SBP readings taken during the first 2 h after waking up) and the lowest nighttime SBP (the average of the three SBP readings centered on the lowest nighttime reading). Analyzing their study group, they discovered a positive connection between the MBPS and participants’ BMI. Yet, when participants were categorized as dippers and non-dippers, this correlation was only significant in the former group [28].

Excess body fat, especially visceral, translating into increased BMI and increased waist circumference, can be responsible for the results observed during the ABPM. Obesity increases insulin and adipocytokines levels and impairs baroreceptors’ sensitivity, enhancing the sympathetic nervous system’s activity [29]. In hypertension, the SNS increases its activity, and vagus nerve cardiac fibers activity decreases [30]. The SNS lowers its activity at night, which causes the blood pressure to dip. Sherwood et al. and Grassi et al. evaluated the relationship between the SNS, SBP dip, and DBP dip, and demonstrated that both dips are significantly related to a decrease in SNS activity [31,32]. In contrast, a sudden increase in sympathetic nervous system activity is responsible for the physiological morning surge in blood pressure. In Chu et al.’s study, they found a significant association between an increase in MBPS and a decrease in the standard deviation of the normal-to-normal RR interval—a measure of cardiac parasympathetic activity—during the 2 h period after waking [28]. During the morning hours, α-sympathetic vasoconstrictor activity rises. Awakening increases epinephrine concentrations, while a postural change elevates both epinephrine and norepinephrine levels. It has been shown that changes in adipocytokine levels may play an important role in the development of hypertension in obesity [14]. Adiponectin protects against insulin resistance, atherosclerosis, and inflammation. It declines due to obesity and hypertension [33]. Low adiponectin predicts aorta stiffness, which leads to an increase in SBP [34]. In a study performed by Vasunta et al., the adiponectin level correlated negatively with daytime SBP, but there was no significant relationship with nighttime SBP or DBP [35]. Obesity is related to a high serum resistin level. Norata et al. observed that resistin correlated positively with SBP but not with DBP [36]. On the contrary, the study conducted by Głuszewska et al. mentioned before indicated significant reductions in leptin and insulin levels alongside a significant decrease in adiponectin levels both ten days and six months after bariatric surgery. There was also a significant decline in the Homeostatic Model Assessment of Insulin Resistance (HOMA-IR). However, no significant alterations in dipping status were observed during these time intervals [27]. There are few studies on the influence of adipocytokines on the blood pressure profile and variability.

Another factor contributing to the differences in blood pressure variability among obese and lean patients is the renin–angiotensin–aldosterone system [37]. It has been observed that excess weight is correlated with higher activity in the renin–angiotensin–aldosterone system both in blood plasma and fat tissue. The high aldosterone-to-renin ratio has been linked to the decrease in the nocturnal blood pressure decline (non-dipping pattern). Satoh et al. reported an excess aldosterone level in comparison to the level of renin, which was linked to higher values of SBP at night and in the morning [38]. However, when considered alone, the plasma aldosterone concentration and renin activity level were not associated with a dipping pattern of hypertension. This correlated with previous studies which stated that hyperaldosteronism is associated with the non-dipping pattern of hypertension. Additionally, in a study by Barochiner et al., which examined the effects of different classes of hypotensive drugs on home blood pressure variability, a statistically significant reduction was specifically observed with aldosterone antagonists [11]. Another study described patients with hypertension and a tumor producing aldosterone; tumor resection led to a greater decrease in blood pressure at night [39]. Moreover, excess aldosterone was related to increased stiffness in the arteries due to the accumulation of collagen in the arterial matrix and fibrosis [40]. The improper vasodilating ability of the arteries of patients with hypertension was described in a 2005 study conducted by Duffy et al. [41]. In this study, patients with hypertension and low renin plasma activity showed an impaired vasodilating response to methacholine and nitroprusside. In the same study, no correlation was shown between renin plasma activity and vasodilating response in normotensive patients.

In addition, many studies have indicated an increased prevalence of abdominal obesity in hypertensive patients and a higher prevalence of hypertension among obese individuals with an increased waist circumference [42,43,44]. Greater waist circumference is associated with increased visceral adiposity and has shown a correlation with the perirenal fat thickness [45]. Additionally, Ricci et al. noticed the potential role of perirenal fat in hypertension among morbidly obese patients (BMI ≥ 40 or ≥35 kg/m^2^ in the presence of comorbidities). Preoperatively, the perirenal fat thickness was higher in hypertensive patients when compared to non-hypertensives 10–12 months after a sleeve gastrectomy. Ricci et al. observed a significant decrease in medication intake during antihypertensive therapy. The decrease in hypotensive therapy was observed only in the group of patients with higher presurgery perirenal fat thicknesses. A significant decrease in the mean waist circumference, SBP, and DBP (17 mmHg for SBP and 11 mmHg for DBP) was observed. This aspect requires further investigations due to previous findings in a meta-analysis in 2013, where there were no significant changes in blood pressure values after bariatric surgery [45,46]. In 12,289 hypertensive adult Caucasians, Chudek et al. observed that obesity, including visceral obesity, impaired the effectiveness of antihypertensive therapy more in males than in females. Of patients exceeding the cutoff values for waist circumference (the same as in our study), 71.3% were hypertensive. In patients with uncontrolled hypertension (SBP and DBP higher than the upper normal limit), nearly 48% presented with a waist circumference greater than cutoff values. Moreover, Chudek et al. noticed that there was a higher predictive value of uncontrolled hypertension for obesity than for visceral obesity assessed with waist circumference. Regardless of gender, blood pressure in individuals with a body weight exceeding 80 kg exhibited poorer control, with a more pronounced effect observed in males. Additionally, among patients with a BMI ≥ 35 kg/m^2^ and a body weight equal to or above 125 kg for males and 110 kg for females, the risk of uncontrolled hypertension was extremely high. It is worth noting that this heightened risk was similar regardless of gender [47]. Numerous studies have elucidated the correlation between the management of hypertension, evaluated through the mean value of several measurements, and waist circumference. However, a dearth of investigations exists that specifically explore the variability of blood pressure in relation to waist circumference.

Another plausible reason for the observed differences in blood pressure variability is undiagnosed obstructive sleep apnea in patients with a higher BMI and in the group with higher waist circumference. In their study, Peppard et al. showed a higher prevalence of sleep-disordered breathing in adults over the decades, which is linked to the ongoing obesity epidemic [48]. At the same time obstructive sleep apnea is often not diagnosed [49]. The link between obstructive sleep apnea and hypertension has been proven in several studies [50]. It is known that obstructive sleep apnea predisposes to the development of hypertension, and on the other hand, it is more common among hypertensive individuals [51]. Crinion et al. conducted a comprehensive study aimed at exploring the relationship between obstructive sleep apnea, blood pressure, and the efficacy of continuous positive airway pressure therapy on blood pressure patterns. Their investigation revealed the prevalence of the non-dipping pattern among patients exhibiting the most severe sleep apnea symptoms. Patients diagnosed with obstructive sleep apnea, when subjected to continuous positive airway pressure therapy, demonstrated a notable improvement in their nocturnal SBP status. Furthermore, continuous positive airway pressure therapy exhibited a positive impact by enhancing the overall blood pressure dip. Their study suggests that the observed non-dipping pattern in hypertension may be linked to oxygen desaturation. It further implies that continuous positive airway pressure therapy, by addressing oxygen desaturation, holds promise as a potential mechanism for improving the non-dipping status of hypertensive patients with sleep apnea [52]. The findings of Pinilla et al. also demonstrated a correlation between obstructive sleep apnea and a non-dipping blood pressure pattern. They identified a metabolic profile in patients with sleep apnea, predominantly characterized by glycerophospholipids, sterols, and glycerolipids. The authors suggested that these lipid classes may exert an influence on blood pressure patterns through oxidative-stress related pathways [53]. In addition, Portaluppi et al., in a study of 100 men with newly diagnosed hypertension, found that non-dipper status and increased blood pressure variability were associated with sleep-disordered breathing [54]. Excess body weight is a well-documented factor in obstructive sleep apnea. Being overweight or obese predisposes patients to the development of obstructive sleep apnea, whereas losing weight reduces the obstructive sleep apnea severity in affected patients [55]. In a study where blood pressure variability was expressed as the standard deviation of the mean values obtained from 24 h blood pressure monitoring of SBP and DBP, the variability was higher in patients with severe obstructive sleep apnea than in those with mild-to-moderate sleep apnea [56]. Furthermore, Xiao Ke et al. showed that patients with essential hypertension, who were newly diagnosed with obstructive sleep apnea, compared to those without such diagnosis, had a higher BMI and a higher 24 h SBP variability, but they did not differ statistically in the DBP variability [57]. On the other hand, in a similar study, Shao et al. found significantly higher 24 h diurnal and nocturnal DBP standard deviation [58]. The common pathophysiological basis of obstructive sleep apnea and hypertension is complex and includes sympathetic tone, the renin–angiotensin–aldosterone system, endothelial dysfunction, and altered baroreceptor reflexes [50] and overlaps with the pathophysiological mechanisms described above.

Several studies highlight variations in the effectiveness of antihypertensive drugs in mitigating short-term blood pressure variability. Monotherapy with calcium channel blockers or diuretics shows reduced blood pressure variability compared to beta blockers, angiotensin-converting-enzyme inhibitors, or angiotensin II receptor blockers. Specifically, within the calcium channel blocker class, amlodipine is associated with lower blood pressure variability than other dihydropyridines. Conversely, when evaluating diuretics like hydrochlorothiazide, chlorthalidone, or indapamide, no discernible differences were observed among them in reducing short-term blood pressure variability. Furthermore, in the management of resistant hypertension, non-pharmacological approaches such as renal denervation also demonstrate a beneficial effect in reducing short-term blood pressure variability [59].

One limitation of this study is the lack of evidence for the causes of the phenomena we found and described. We can only hypothesize about the mechanisms that may be involved, and more mechanistic studies should be carried out. We support the hypothesis that some hormonal changes are more responsible for this phenomenon than obstructive sleep apnea because, in the studied population, we did not find a relationship between the blood pressure variability and values and sleep disorder derivatives like sleepiness or the Eppworth scale. In addition, as this is only a cross-sectional study, we cannot make any statements about the role of these phenomena in terms of the prognosis of the patients. However, this observation leads to simple clinical conclusions. A different approach to the evaluation of hypertension in obese patients should be considered, taking into account the nocturnal variability of blood pressure. Another limitation is that we analyzed data both from newly diagnosed and on-treatment patients. The premise of creating such a study group was to analyze data from real life population. Thus, the results obtained can be applied to the clinical decisions in many patients.

## 5. Conclusions

By analyzing the changes in blood pressure hour by hour, we observed the dawn phenomenon, a significantly higher SBP and its variability, in the last hours before waking in obese individuals in comparison to the non-obese ones. This difference is due to a smaller nocturnal decrease in these values in the obese patient groups than in the non-obese group. Finding the causes and consequences of this phenomenon requires further research. However, the obtained results indicate the necessity of an individualized approach to the treatment of arterial hypertension in obese patients.

## Figures and Tables

**Table 1:** General characteristics of the group: data are presented as the number and percentage of the study population or the mean value among the study population.

Parameters	Study Group	
N	1345	
Age [Years]	56 ± 15	
Sex	Female	Male
	644 (48%)	701 (52%)
Body mass index [kg/m^2^]		
<25	355 (26%)	
≥25 and <30	550 (41%)	
≥30 and <35	292 (22%)	
≥35	148 (11%)	
Waist circumference [cm]		
Lower (F < 88, M < 102)	671	
Higher (F ≥ 88, M ≥ 102)	674	
Office BP [mmHg]		
Systolic	140 ± 22	
Diastolic	82 ± 13	
Nighttime BP [mmHg]		
Systolic	116	
Diastolic	68	
Daytime BP [mmHg]		
Systolic	138	
Diastolic	83	

BP: blood pressure; F: female; M: male.

**Table 2:** Concomitant diseases in patients from each BMI group.

	BMI < 25 kg/m^2^	25 ≤ BMI < 30 kg/m^2^	30 ≤ BMI < 35 kg/m^2^	BMI ≥ 35 kg/m^2^
type 2 diabetes	2%	7%	12%	27%
CAD	5%	7%	10%	16%
pre-diabetes	4%	7%	10%	12%
CKD	9%	10%	10%	16%
stroke	4%	2%	4%	1%

CAD: coronary artery disease; CKD: chronic kidney disease.

**Table 3:** Hypotensive medications used by patients from each BMI group.

	BMI < 25 kg/m^2^	25 ≤ BMI < 35 kg/m^2^	30 ≤ BMI < 35 kg/m^2^	BMI ≥ 35 kg/m^2^
ACE-i	38%	40%	45%	47%
ARB	14%	20%	25%	27%
diuretics	24%	33%	46%	52%
BB	32%	37%	48%	56%
CCB	31%	34%	43%	42%
MRA	3%	3%	5%	10%
number of drugs [mean]	1.4	1.7	2.1	2.3

ACE-i inhibitors of angiotensin converting enzyme; ARB: angiotensin II receptor blockers; BB: beta-blockers; CCB: calcium channel blockers; MRA: mineralocorticoid receptor antagonists.

## Data Availability

Data sets generated and analyzed during the current study are available from the corresponding author upon reasonable request.
